# “It was God’s will”: Continuing pregnancy after perinatal infection by Zika virus*

**DOI:** 10.1590/1518-8345.3485.3310

**Published:** 2020-08-31

**Authors:** Celmira Laza-Vásquez, Keila Vanessa Cortés-Martínez, Juan Pablo Cano-Rivillas

**Affiliations:** 1 Universitat de Lleida, Facultad de Enfermería y Fisioterapia, Lleida, Cataluña, Spain.; 2 Universidad Surcolombiana, Programa de Enfermería, Neiva, Huila, Colombia.

**Keywords:** Decision Making, Pregnancy, Zika Virus Infection, Microcephaly, Religion, Qualitative Research, Tomada de Decisões, Gravidez, Infecção por Zika virus, Microcefalia, Religião, Pesquisa Qualitativa, Toma de Decisiones, Embarazo, Infección por el Virus Zika, Microcefalia, Religión, Investigación Cualitativa

## Abstract

**Objective:**

to understand the influence of the religious beliefs on the decision of a group of women residing in the Huila Department to continue their pregnancies despite perinatal infection by the Zika virus.

**Method:**

a focused ethnography. The participants were 21 women who had presented a perinatal infection by the Zika virus and whose babies were born with congenital microcephaly. 2 discussion groups and 6 semi-structured interviews were conducted, and thematic analysis was used for data treatment.

**Results:**

three themes emerged, namely: “God, why me?” is the initial questioning of the women to God for the prenatal diagnosis of microcephaly in their babies, “Clinging to a divine miracle” describes how the women did not lose their faith and begged for a divine miracle for their babies to be born healthy, and “It was God’s will” means acceptance, resignation, and respect for God’s will, as well as the denial to abort despite the medical recommendations.

**Conclusion:**

religiosity and religious beliefs were determinant factors in the women’s decision to continue their pregnancies. It becomes necessary to continue investigating this theme to understand their experiences and to generate follow-up and support actions from nursing care.

## Abstract (pt)

**Objetivo:**

compreender a influência das crenças religiosas na decisão de um grupo de mulheres residentes no Departamento de Huila de continuar a gestação apesar da infecção perinatal pelo vírus Zika.

**Método:**

etnografia focalizada. Participaram 21 mulheres que haviam apresentado infecção perinatal pelo vírus Zika, cujos filhos nasceram com microcefalia congênita. Foram realizados 2 grupos de discussão e 6 entrevistas semi-estruturadas e foi utilizada a análise temática para o tratamento dos dados.

**Resultados:**

três temas surgiram: “Deus: por que comigo?” é um questionamento inicial das mulheres a Deus devido ao diagnóstico pré-natal de microcefalia do(a) filho(a), “Apegar-se a um milagre divino” descreve como as mulheres não perderam a fé e imploraram por um milagre divino para que seus filhos nascessem sadios e “Era a vontade divina” significa a aceitação, resignação e respeito pela vontade divina e a negação a abortar, apesar das recomendações médicas.

**Conclusão:**

a religiosidade e as crenças religiosas foram determinantes na decisão das mulheres de continuar a gestação. Faz-se necessário continuar a indagação sobre esse tema, para compreender as experiências dessas mulheres e criar ações de acompanhamento e apoio a partir dos cuidados de enfermagem.

## Abstract (es)

**Objetivo:**

comprender la influencia de las creencias religiosas en la decisión de un grupo de mujeres residentes en el Departamento de Huila de continuar sus embarazos a pesar de la infección perinatal por el virus del Zika.

**Método:**

etnografía focalizada. Participaron 21 mujeres que habían presentado una infección perinatal por el virus del Zika y cuyos hijos nacieron con microcefalia congénita. Se realizaron 2 grupos de discusión y 6 entrevistas semi-estructuradas y se utilizó el análisis temático para el tratamiento de los datos.

**Resultados:**

surgieron tres temas: “Dios: ¿Por qué a mí?” es el cuestionamiento inicial de las mujeres a Dios por el diagnóstico prenatal de microcefalia de sus hijos(as), “Aferrarse a un milagro divino” describe cómo las mujeres no perdieron la fe e imploraron por un milagro divino para que sus hijos nacieran sanos, y “Era la voluntad divina” significa la aceptación, resignación y respeto por la voluntad divina y la negación a abortar a pesar de las recomendaciones médicas.

**Conclusión:**

la religiosidad y las creencias religiosas fueron factores determinantes en la decisión de las mujeres de continuar sus embarazos. Se hace necesario profundizar la investigación en este tema para comprender sus experiencias y generar acciones de acompañamiento y apoyo desde el cuidado de Enfermería.

## Introduction

In October 2015, Colombia declared an outbreak of the Zika virus disease, and the Huila Department presented the third highest accrued incidence of the country (517 *per* 100,000 inhabitants) and in pregnant women (333 *per* 100,000 inhabitants)^(^ 1 ^)^. By January 2018, 248 cases of congenital syndrome related to perinatal infection by the Zika virus have been confirmed in the country^(^ 2 ^)^.

Perinatal infection by the Zika virus has been related to adverse pregnancy results, especially microcephaly and other severe brain anomalies like intellectual disability, ophthalmologic and auditory alterations and epilepsy^(^ 3 ^-^ 4 ^)^.

A diagnosis of fetal congenital malformation means intense pain and emotional shock to the mothers due to the prenatal attachment^(^ 5 ^)^. Multiple losses and profound feelings of worry are also generated in the parents, and those who decide to continue their pregnancies not only experimented the loss of a healthy child and its anticipated future, but also that of a happy pregnancy^(^ 6 ^)^. In the case of a pregnancy with a diagnosis of congenital malformation due to perinatal infection by the Zika virus, the parents’ future becomes uncertain; with material evidence of primary reactions of anguish, guilt, indecision and shame; then to experience a negotiation, acceptance, and adaptation reaction to the new condition^(^ 7 ^)^.

In the city of Neiva, a specific fact was the decision of a group of mothers who decided not to interrupt their pregnancies, despite having been explained the conditions in which their babies would be born and the impact on the quality of life of both the infants and the families. The aforementioned is to be interpreted considering the option of legal abortion in case of fetal malformations offered by the 2006 C-355 Sentence^(^ 8 ^)^ and by the World Health Organization’s recommendation to provide access to a secure abortion in case of suspected or confirmed microcephaly due to perinatal infection by the Zika virus^(^ 9 ^)^.

The objective of this study is to understand the influence of religious beliefs on the decision of a group of women residing in the Huila Department to continue their pregnancies despite the perinatal infection by the Zika virus.

## Method

The design chosen was that of a focused ethnography, an option through which it is sought to pay attention to the small elements and activities in which people get involved, which is particularly helpful to obtain information on a specific theme and whose object of study is limited to small social groups. Focused ethnographies are characterized by focused research questions, short-term field visits, and intensity in data collection and analysis^(^ 10 ^)^.

The study was conducted during 2018 in the city of Neiva, the capital and most important municipality of the Huila Department (Southern Colombia). Due to the geographical and climatic factors of this region, there is circulation of the Zika virus^(^ 11 ^)^. The participants were 21 women who presented perinatal infection by the Zika virus between 2015 and 2016, and whose babies were born with congenital microcephaly and other neurological alterations related to this infectious event. All the 21 participants are members of a support group and of the “*Hijos del Zika: Milagro de Dios*” (“Zika Children: God’s Miracle”) Association.

All the women were of age at the time of data collection and voluntarily wished to participate in the study. The group presented an age range between 18 and 37 years old (with a mean of 25 years old). Only two of them had University schooling and four just elementary school; 18 declared to be unemployed since their babies had been born and six were single. 19 declared themselves Catholics, and 2 active Christians (Table 1).

**Table 1:** Characteristics of the women (n=21) participating in the study. Neiva, Huila, Colombia, 2018

P*	SGSSS† membership	Age of the women	Age of the child||	Schooling level	Occupation	Marital status	No. of children
1	‡C	29	23	Technical	First childhood teacher	Consensual union	2
2	§C	30	27	University	Kindergarten teacher	Married	1
3	S	18	27	Elementary school	Unemployed	Single	1
4	C	24	14	High school	Unemployed	Married	1
5	S	20	24	Elementary school	Unemployed	Consensual union	1
6	C	36	28	High school	Unemployed	Married	4
7	C	21	24	High school	Unemployed	Single	1
8	S	19	24	High school	Unemployed	Consensual union	1
9	C	19	27	High school	Unemployed	Consensual union	1
10	C	20	24	High school	Unemployed	Single	1
11	S	37	28	University	Unemployed	Married	3
12	S	31	29	High school graduate	Unemployed	Consensual union	1
13	S	23	26	Technical	Unemployed	Single	1
14	S	25	22	High school graduate	Hair stylist	Single	2
15	S	25	24	High school	Unemployed	Married	2
16	S	30	26	High school	Unemployed	Single	3
17	S	19	24	Elementary school	Unemployed	Consensual union	1
18	C	24	28	Technical	Unemployed	Consensual union	2
19	S	24	18	Technical	Unemployed	Consensual union	1
20	S	23	36	High school	Unemployed	Consensual union	1
21	S	30	24	Elementary school	Unemployed	Single	4

* P = Participants;

† SGSSS = Membership to the General Social Security System in Health (*Sistema General de Seguridad Social en Salud*);

‡ C = Contributive;

§ S = Subsidized;

|| Age in months

The first step of the research was contacting each of the leaders who served as field gatekeeper^(^ 12 ^)^ for entering the group, the person in charge of inviting the women to participate in the study, invitation accepted by all. Towards the middle of 2018, the research group met once a month on six occasions with the women to create and strengthen the women’s support group. Through various group activities, the support group meetings focused on the women’s statements about their experiences during pregnancy, birth, upbringing, and care for their children. Thus, it turned into a personal space to express and share fears and worries with other women who were facing similar experiences; and to build a group identity which was structured around a “communion” instead of around simple understanding^(^ 13 ^)^. Not to mention establishing a strong bond between the researches and the women. In this space, data was collected between September and December 2018.

Data collection was performed by the lead researcher, and two discussion groups and six one-on-one interviews were used. The first technique facilitates deep exploration of the information through perceptions, experiences, and attitudes of participants selected for sharing similar experiences or characteristics. Its conversational nature allows for a dynamic interaction and synergy among the participants, producing very rich data^(^ 14 ^-^ 15 ^)^. Data collection was started with this technique to perform an initial exploration of the theme, of which there is little knowledge on the scientific literature^(^ 15 ^)^.

The discussion groups lasted between 60 and 90 minutes, and were guided by the following questions: Tell us what you felt when you were told that your child might be born with microcephaly and How did your religious beliefs influence on your decision to continue your pregnancy? The discussion and discourse construction were generated by the participants.

Additionally, six semi-structured interviews were conducted^(^ 16 ^)^, each with a mean duration of 90 minutes, to expand, deepen, and clarify the theme which emerged in the discussion groups. The questions presented in the interviews emerged from the analysis of the discussion groups and were made until no new data was obtained or which allowed deepening on the study phenomenon, that is to say, until reaching information saturation^(^ 16 ^)^.

Data collection was performed in the Medical School of Universidad Surcolombiana. Data collection and analysis were performed parallelly. The discussion groups and interviews were audio-recorded and transcribed in the following 48 hours by the research group, post-anonymization.

Thematic analysis^(^ 17 ^)^ was used for treating the data; this is a method to identify, analyze, and report patterns (themes) inside the data, and which is adapted to a wide range of research interests and theoretical perspectives. This included familiarization with the data, coding, search for themes, review of themes, and definition and naming of themes.

Data analysis was performed by the three researchers, by separately reading the transcriptions of the discussion groups and of the interviews, and by listening the recordings when doubts or relevant information arose in the content of the transcriptions to familiarize with the data. Subsequently, the coding step was started by means of a new reading of the transcriptions, labelling the relevant data, and excluding those that were not related to the objective set out. Once the data of interest were labelled, the search was started for significant patterns through the identification of similarities in the coded data, with the identification of four themes. After a new review and discussion of the codes, they were synthesized into three, which were named and organized considering the meaning of the participants’ statements. The final step was writing the themes, which implied weaving the analytical narrative and the verbatim wordings to account for them in a coherent and logical way. The ATLAS.ti 8 program was used as support for data analysis.

Once the results were elaborated, the participants were invited to their review and they were accepted with no further suggestions.

During the research process, the methodological rigor criteria^(^ 18 ^)^ were observed to safeguard the methodological quality of the research: credibility, auditability, and transferability. All the ethical aspects set out by the 1993 Colombian Resolution 8430^(^ 19 ^)^ were taken into account. The support of the Research Ethics Committee of the Medical School of Universidad Surcolombiana (Minutes 003/20, of April 2018) was obtained, data confidentiality and the participants’ anonymity were guaranteed, and all the participants signed the Informed Consent.

## Results

Three themes emerged from the statements, through which the women accounted for their decision to continue their pregnancies: “God, why me?”, “Clinging to a divine miracle”, and “It was God’s will”.

*“God, why me?”* The initial diagnosis became a painful and disturbing news which the women asserted they were not prepared for. Hearing the term “microcephaly” for the first time disconcerted them, as well as the conditions in which their children would live if they were born. Below is how one of them narrates it: *(…) I arrived normal, glad, I was even happy because I was going to see the baby again in the echography (…) when that face of the doctor, he made like a sad face… My doc: What happened? The he said: “Wait on me a little” (…). “Mommy, possible microcephaly” and I said: Microcephaly? I didn’t know anything! And me: doctor, And what is that? Well I looked at him in the face and said: No, that’s something bad! (…) I got cold as ice, with that sensation like of crying. And of course, when he explains to me that it is brain damage, that the baby had a two-week delay in the head* (Discussion group P3).

The news destroyed the women’s dreams of a healthy child. Between pain and guilt, only at first did they wonder if they had done anything wrong in their lives and demanded to their God why this was happening to them if they had always behaved correctly. They also considered that it was unfair because they wished that baby and had the necessary conditions to take care of it. Added to this were other events in their personal lives like the craving for that first child or for the first son, and the loss of other pregnancies and the wait and preparation time for a new pregnancy.

*At first I said: But, why me? What did I do? I always try to do things right… And I said: What have I done? A year before I had lost a 5 month baby girl, due to previous placenta, so I already had a bigger sorrow which is losing a baby, and for me getting this other case “for free”…*(Discussion group P7).

*“Clinging to a divine miracle.”* Despite confirmation of the diagnosis, the women did not lose their faith. On the contrary, they clung to God and begged for a miracle so that their babies were born healthy and without microcephaly; and, if that was not possible, that they did not have any other congenital malformations or were stillborns: *(…) I started clinging to faith… There is a God and indeed miracles exist!* (Discussion group P1).

With the hope of a miracle, they reasserted and strengthened their faith in God, and stayed optimistic that the initial diagnosis was a mistake. This led them and their families to develop various religious practices: attendance to cults, payment of promises, charity actions with those in most need, and search for support with religious leaders.

*(…) I clung to God and to the Virgin… I came closer to two priests, they prayed a lot to my daughter on the belly (…) I prayed, all together, I didn’t stop the rosary not a single day during my pregnancy (…)* (Discussion group P18).

Maintaining alive the hope of a healthy child also responded to a diagnosis at the end of the pregnancy, between the 6^th^ and 8^th^ months, and to the fact that, up to that moment, no diagnostic examination had reported alterations in the fetus. They had already seen their children through echographies and listened to their heartbeats; and they had a strong attachment to them. So they embraced the hope of a possible mistake in the medical diagnosis.

*I went to controls, everything came out right. I went through 3 detailed echographies and everything came out very well (…). Already at 8 months, in the last echography I saw that when it came out, the head was smaller than the body* (One-on-one interview P3).

On the other hand, it was inconceivable for the women to relate the condition their children would be born with as the result of a bite of a mosquito that carried a virus. They found it impossible to understand how something so “small and insignificant” could provoke so much harm: *My God, how is it that an insect? How is it that a mosquito can do so much harm? You barely imagine the things that happen! And you still wonder: How? Why?* (Discussion group P2).

Maintaining alive the hope of a miracle led them not to accept the initial medical diagnosis and, for that reason, they travelled a long and hard road in the search of other answers, different from the initial one, and which confirmed to them that it was a medical error. This implied new examinations and other medical opinions, as well as time, efforts, and economical resources.

*(…) they told me [physicians]: “We’re going to meet for the procedure” Procedure of what? “No, it’s that we’re going to interrupt this pregnancy [physicians]. We’re going to what!? I said: No! And the fight had begun, from one doctor to another. I spent what money I didn’t have going to all the specialists, hoping for a possibility. Who guarantees me that the doctors are not wrong?* (Discussion group P6).

*“It was God’s will.”* After the devastating diagnosis and the search for other different answers they did not obtain, the participants were left only with acceptation, resignation, and respect for God’s will: *(…) it was God’s will it came like that* (Individual interview P4).

It was for this reason that they did not contemplate the option of interrupting their pregnancies: *No one is above God! The only one who has the right to kill his own children is God, he who creates them* (Discussion group P15). This assertion was the strongest argument of the women to reject (and not even consider) the possibility of abortion. Since most of the families were believers, they supported the decision.

For this reason, they did not consider the tough description from physicians, psychologists, and social workers about the conditions in which their children would live: psychomotor delay, health problems caused by other malformations, and convulsive syndrome, among others; problems which would greatly affect the quality of life of the minors. The only thing that mattered was the argument that God was sending the babies to them and that their birth was part of a divine purpose which only He would reveal to them in time, in *God’s time* (One-on-on interview P2).

*(…) they confirmed that we’re in fact in front of a case of microcephaly by the Zika virus. They tell us [physicians] more or less generally what we were facing (…) not only microcephaly, but other possible malformations (…) the chance that she’d be blind, live only 24 hours… even die before being born. That is to say, it was but bad news all the time, only negative things… they didn’t talk about anything positive. And so obviously, we had the right to say if we wanted to interrupt our pregnancy (…) and so obviously my husband and I didn’t have to think at all (…) if God had decided to send her to the world like that, there had to be some purpose for her and for us* (One-on-one interview P2).

In the logic of the divine will discourse, interrupting the pregnancy also meant violating one of the divine mandates: “Thou shall not kill.”

*(…) a doctor who also told me the same thing [interrupting the pregnancy]. Then that day I already got angry and so I told him that I was going to continue, that I was going to have [son], I was going to have him, but that I didn’t want anyone to say that to me again. That if [son] was going to die, let that be because it’s God’s will, not because I was going to kill my son* (One-on-one interview P1).

Despite the aforementioned, the health professionals who cared for them insisted on several occasions on the option to interrupt their pregnancies. In fact, many of the women stated that they felt they were questioning their decision to continue their pregnancies. They also felt pressure by what they considered “too much” insistence and “excessively” crude explanations about the condition in which their children would be born and the health conditions with which they would live; as well as the different personal, economic, and social problems that this would bring about in their lives, and in the lives of their partners and families.

*At six months the psychologist was telling me that he [son] was going to be a vegetable, that if I was going to have another baby I wasn’t going to be able to because I had to devote time to [son] (…) then the only thing I told them was that it was me that was going to care for him (…) that I was never going to call them to help me care for him. I continued my pregnancy (…)* (Discussion group P3).

The decision to continue their pregnancies despite the suggestions not to do so also responded to the women’s argument that this was a decision incumbent only to them, to their partners, and to God. For two of them, another reason was feeling that the pregnancy was an answer from God to their asking for forgiveness for past mistakes, like a previous abortion or not wishing to have a baby.

Finally, they considered that, just as God sent them their children with that condition, He would also send them the economic, emotional, and human means for their care and support. For this reason, the women were willing to make any kind of sacrifice for the child that God was giving them.

## Discussion

For most of the couples, pregnancy is a happy experience for waiting a healthy child. A late diagnosis of a fetal anomaly is infrequent, something unexpected and traumatic and the women who experience it find the event extremely difficult. It also sets out a challenge in the decision making process to continue the pregnancy, especially when there is fetal viability (≥24 weeks)^(^ 20 ^)^.

The scientific literature shows that the discovery of a fetal malformation is shocking and painful both for the women and for their partners; and deciding to interrupt the pregnancy is a complex and difficult process implying multiple factors which surpass the medical diagnosis. Unfortunately, most of the studies on the theme come from developed countries with economic and social conditions which differ from the Colombian context, and where abortion is socially accepted. These studies evidence that the most frequent decision is to interrupt the pregnancy, decision for which the women search for other medical opinions, those of their relatives and of parents of children with the same diagnosed fetal anomaly. The decision is supported by the worries about the future, the type of disability, quality of life, and the possibility of the child suffering after being born; as well as the burden which caring for the child would imply for their families^(^ 6 ^,^ 21 ^-^ 26 ^)^.

However, the situation is different in the Colombian context, and a possible answer is the strong presence of the Catholic Church and of several Evangelic Churches of different denominations. This is a central element to understand the decision of the study participants to continue their pregnancies despite evidence of microcephaly, the recommendations from the health professionals not to do so, and the legal option to interrupt their pregnancies. Thus, the women’s statements accounted for the weight of religiosity and of their religious beliefs on their decisions.

Religion allows people to assign a meaning to their beliefs, experiences, and practices in different life situations^(^ 27 ^)^ and religiosity is a means for the individuals to express their spirituality through the adoption of values, beliefs, and ritual practices which answer the main questions about life and death^(^ 28 ^)^. On their own, religious beliefs can help people to come to an agreement on the different problems in their lives and, frequently, they entail accepting adversity with the guidance of a bigger force which directs life itself^(^ 29 ^)^.

Several authors explain that, although they are difficult for people to define, the meanings of religiosity and of religious beliefs are necessary, help to understand the meaning of life and to find a balance in it, and that they are a strength and resource to face crisis^(^ 28 ^)^. Thus, these two aspects are configured as strong determinants to continue the pregnancy despite the results of a diagnosis of a prenatal congenital malformation, given the religious prohibition to interrupt pregnancies, religious fatalism as a reason to continue them, and the sensation of help which religious beliefs offer to the women to stay optimistic with respect to their pregnancies and to the health of their child to be born^(^ 30 ^)^.

A scarce number of studies have documented religiosity and religious beliefs as determinants in women and their partners to continue their pregnancies despite a diagnosis of fetal congenital malformations. This is influenced by the religious prohibition to interrupt pregnancies, as a synonym of killing and as a generator of guilt in women; the belief of children as a gift from God, the conviction that the child to be born’s death must be natural and not provoked, the recognition of the fetus as a person, and the hope for a cure of the anomaly^(^ 5 ^,^ 20 ^,^ 30 ^)^. In Colombia, the abortion option generates conflicts in women because of the worries about the destiny of the souls of embryo/fetus and of the women, turning it into a synonym of killing, of sin, of condemnation to hell, and of stigma^(^ 31 ^)^.

In turn, religiosity and religious beliefs turn into a coping mechanism in the face of difficult situations and soften the effects of stress on life, increasing self-esteem or the sense of self-efficacy and openness to social support^(^ 32 ^)^.

Despite the limitations of this study lying on the size of the sample, on the non-inclusion of the women’s partners and of the health professionals in the sample, and on the fact that the results are circumscribed to a region of Colombia, as far as we know, it is the first one that incorporates the voices of the women who decided to continue their pregnancies when faced with a diagnosis of congenital microcephaly related to perinatal infection by the Zika virus, thus helping to understand the complexity and the challenges faced by the women.

It also helps to understand the weight of religiosity and of religious beliefs on the decision making of people with respect to the processes related to health, disease, and care practices. This knowledge is indispensable for the nurses to provide care from a holistic perspective of the care subjects. It is also indispensable that this knowledge implies greater understanding of the spiritual dimension and its importance for health. In this sense, they draw the attention to the need of considering these two categories in the educational, investigative, and assistential work in Nursing.

## Conclusion

This study shows that religiosity and religious beliefs were determinant factors in the participants’ decision to continue their pregnancies when faced with a congenital malformation like microcephaly related to infection by the Zika virus during pregnancy. All despite the information and recommendations offered by the health professionals who cared for them, and in view of the possibility of legally interrupting their pregnancies. The women’s decision was supported by their families.

It is necessary to continue with the research efforts to deepen on the influence of religiosity and religious beliefs on health in different social contexts and groups of people.

The results of this study are circumscribed to the context of a Colombian region, reason for which future studies are needed in different Latin American regions where births of children with microcephaly have been reported during the 2015 and 2016 epidemics. Likewise, on other themes pertaining to this particular collective of women like the upbringing and care experiences and the ways to cope with the problems derived from giving birth to their children; which will allow us to understand the experiences and to generate actions for follow-up and support from nursing care.
